# Association between dietary macronutrient composition and plasma one-carbon metabolites and B-vitamin cofactors in patients with stable angina pectoris

**DOI:** 10.1017/S0007114524000473

**Published:** 2024-05-28

**Authors:** Marianne Bråtveit, Anthea Van Parys, Thomas Olsen, Elin Strand, Ingvild Marienborg, Johnny Laupsa-Borge, Teresa Risan Haugsgjerd, Adrian McCann, Indu Dhar, Per Magne Ueland, Jutta Dierkes, Simon Nitter Dankel, Ottar Kjell Nygård, Vegard Lysne

**Affiliations:** 1 Mohn Nutrition Research Laboratory, Department of Clinical Science, University of Bergen, Bergen, Norway; 2 Centre for Nutrition, Department of Clinical Science, University of Bergen, Bergen, Norway; 3 Department of Nutrition, Institute of Basic Medical Sciences, University of Oslo, Oslo, Norway; 4 Department of Immunology and Transfusion Medicine, Haukeland University Hospital, Bergen, Norway; 5 Department of Global Public Health and Primary Care, University of Bergen, Bergen, Norway; 6 Bevital AS, Bergen, Norway; 7 Centre for Nutrition, Department of Clinical Medicine, University of Bergen, Bergen, Norway; 8 Laboratory Medicine and Pathology, Haukeland University Hospital, Bergen, Norway; 9 Department of Heart Disease, Haukeland University Hospital, Bergen, Norway

**Keywords:** B-vitamins, FFQ, Macronutrients, Metabolomics, One-carbon metabolism

## Abstract

Elevated plasma concentrations of several one-carbon metabolites are associated with increased CVD risk. Both diet-induced regulation and dietary content of one-carbon metabolites can influence circulating concentrations of these markers. We cross-sectionally analysed 1928 patients with suspected stable angina pectoris (geometric mean age 61), representing elevated CVD risk, to assess associations between dietary macronutrient composition (FFQ) and plasma one-carbon metabolites and related B-vitamin status markers (GC–MS/MS, LC–MS/MS or microbiological assay). Diet-metabolite associations were modelled on the continuous scale, adjusted for age, sex, BMI, smoking, alcohol and total energy intake. Average (geometric mean (95 % prediction interval)) intake was forty-nine (38, 63) energy percent (E%) from carbohydrate, thirty-one (22, 45) E% from fat and seventeen (12, 22) E% from protein. The strongest associations were seen for higher protein intake, i.e. with higher plasma pyridoxal 5’-phosphate (PLP) (% change (95 % CI) 3·1 (2·1, 4·1)), cobalamin (2·9 (2·1, 3·7)), riboflavin (2·4 (1·1, 3·7)) and folate (2·1 (1·2, 3·1)) and lower total homocysteine (tHcy) (–1·4 (–1·9, −0·9)) and methylmalonic acid (MMA) (–1·4 (–2·0, −0·8)). Substitution analyses replacing MUFA or PUFA with SFA demonstrated higher plasma concentrations of riboflavin (5·0 (0·9, 9·3) and 3·3 (1·1, 5·6)), tHcy (2·3 (0·7, 3·8) and 1·3 (0·5, 2·2)) and MMA (2·0 (0·2, 3·9) and 1·7 (0·7, 2·7)) and lower PLP (–2·5 (–5·3, 0·3) and −2·7 (–4·2, −1·2)). In conclusion, a higher protein intake and replacing saturated with MUFA and PUFA were associated with a more favourable metabolic phenotype regarding metabolites associated with CVD risk.

Several metabolite markers have been associated with risk of CVD, including one-carbon metabolites such as total homocysteine (tHcy)^(1)^, methylmalonic acid (MMA)^(2–4)^, dimethylglycine (DMG)^(5–7)^, cystathionine^(8–10)^ and choline^(7,11–14)^. One-carbon metabolism comprises all metabolic reactions involving the transfer of one-carbon units and includes the methionine-homocysteine cycle, the transsulfuration pathway, the folate cycle and the choline oxidation pathway (Fig. 1). Changes in one-carbon metabolites may result from altered metabolic states in different tissues, which in turn may depend on dietary intake of energy-yielding nutrients. More specifically, protein restriction in both healthy subjects, as well as in subjects with inborn errors of the metabolism of sarcosine, leading to elevated plasma concentrations of sarcosine, increased the remethylation of homocysteine to methionine^(15,16)^. Moreover, inverse associations with plasma tHcy were reported for a higher protein intake, as well as for intakes of fish and eggs^(17)^. Total protein intake was also reported to be positively associated with plasma cystathionine and total cysteine, and a higher intake of plant protein in particular was reported to be inversely associated with tHcy^(18)^. Some studies also suggest that different protein sources may elicit opposite effects on plasma tHcy, as they report diets high in plant protein to be inversely associated with tHcy and diets high in animal protein to be positively associated with tHcy^(19,20)^.

**Fig. 1.**
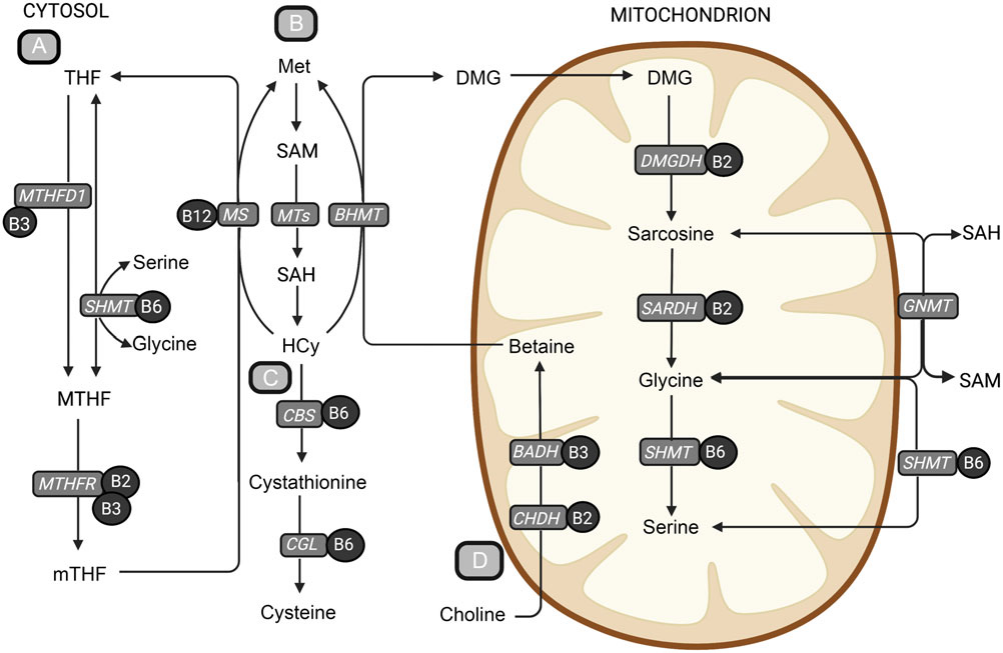
An overview of central metabolic pathways in one-carbon metabolism. (a) The folate cycle, (b) the methionine-homocysteine cycle, (c) the transsulfuration pathway and (d) the choline oxidation pathway. The metabolites are shown in bold text, and B-vitamin cofactors are shown in black circles. The enzymes are presented in grey boxes. Methionine is an important precursor to the central methyl donor S-adenosylmethionine. When S-adenosylmethionine donates a methyl group, it is converted to SAH, which is hydrolysed to homocysteine. Homocysteine can be further remethylated back to methionine or go through the irreversible transsulfuration pathway forming cystathionine and cysteine. The remethylation of homocysteine back to methionine is dependent on the donation of a methyl group and can occur in two ways. The folate-dependent remethylation pathway uses 5-methyltetrahydrofolate as the methyl donor and is catalysed by the vitamin B_12_-dependent enzyme, methionine synthase, generating methionine and tetrahydrofolate. Tetrahydrofolate can go through the folate cycle again to form 5-methyltetrafolate, which again can be used in the remethylation of homocysteine. The second homocysteine remethylation pathway uses betaine from the choline oxidation pathway as the methyl donor, forming methionine and DMG, catalysed by betaine-homocysteine methyltransferase (*BHMT*). DMG can then be further demethylated in the mitochondrion, forming sarcosine, glycine and serine through several enzymatic reactions using B-vitamins as cofactors. BADH, betaine aldehyde dehydrogenase; BHMT, betaine-homocysteine methyltransferase; CBS, cystathionine-β-synthase; CGL, cystathionine-*γ*-lyase; CHDH, choline dehydrogenase; DMG, dimethylglycine; DMGDH, dimethylglycine dehydrogenase; GNMT, glycine-N-methyltransferase; Hcy, homocysteine; Met, methionine; MS, methionine synthase; MTHF, 5,10-methylenetetrahydrofolate; MTHFD1, methylenetetrahydrofolate dehydrogenase complex 1; MTHFR, methylenetetrahydrofolate reductase; MT, methyltransferases; mTHF, 5-methyltetrahydrofolate; SAH, S-adenosylhomocysteine; S-adenosylmethionine, S-adenosylmethionine; SARDH, sarcosine dehydrogenase; SHMT, serine hydroxymethyltransferase; THF, tetrahydrofolate. Created with BioRender.com.

Food sources of protein are commonly good sources of vitamin B_6_, and protein intake is positively related to vitamin B_6_ status^(21)^. Dietary PUFA have also been reported to be inversely associated with plasma tHcy^(17)^ and randomised controlled dietary intervention trials with increasing PUFA intakes and/or altered intakes of methionine and cysteine affected plasma concentrations of several metabolites related to transmethylation, transsulfuration and B-vitamin status^(22,23)^. Further, supplementation with krill oil, which is rich in phosphatidylcholine and *n*-3 fatty acids, reduced tHcy and increased the concentration of choline oxidation pathway metabolites in healthy adults^(24)^. In rats, increasing dietary fat intake has been shown to upregulate genes involved in the choline oxidation pathway and to downregulate both enzymes of the transsulfuration pathway^(25)^. Further, when combined with methionine restriction, betaine induced betaine-homocysteine methyltransferase *(BHMT)* mRNA in rats^(26)^. For choline, dietary sources include eggs, milk, lean fish and leafy vegetables, and dietary total choline has moreover been reported to be positively associated with plasma choline, methionine, cystathionine, cysteine and DMG and inversely associated with plasma tHcy, glycine and serine^(27,28)^. Total carbohydrate intake is also positively associated with circulating tHcy concentrations, while the opposite has been seen for vegetables and whole grain^(17)^. Indeed, whole-grain cereals are a main source of betaine^(29)^, and whole-grain intake has been associated with higher plasma betaine concentrations^(30)^.

Taken together, there is an established connection between one-carbon metabolites and CVD and evidence implicating a role of diet in the regulation of these metabolic pathways. This underscores the relevance of our study, where the aim was to explore associations between dietary macronutrient composition and plasma concentrations of one-carbon metabolites and associated B-vitamin status markers. To deepen our understanding of the interplay between diet and one-carbon metabolism, we here leverage a large cohort of patients with stable angina pectoris who are at increased CVD risk and where both dietary and metabolite data are available.

## Methods

### Study population

This cross-sectional study utilises data from the Western Norway B-vitamin Intervention Trial consisting of 3090 participants randomised to receive tHcy-lowering B-vitamins (Clinical Trials Identifier NCT00354081)^(31)^. The source population for Western Norway B-vitamin Intervention Trial was patients referred to coronary angiography for suspected coronary artery disease between 2000 and 2004. The analyses in the present study only include patients diagnosed with stable angina pectoris (*n* 2573).

Participants filled out an FFQ at baseline. Participants were excluded if they did not complete the FFQ (*n* 485), left more than one page blank (*n* 80) or reported very high (> 15 000 kJ/d (> 3585 kcal/d) for women and > 17 500 kJ/d (> 4182 kcal/d) for men) or low (< 3000 kJ/d (< 717 kcal/d) for women and < 3300 kJ/d (< 788 kcal/d) for men) total energy intake (*n* 27) to improve accuracy of the dietary data and account for potential misreporting as well as under- and overreporting. The cut-offs we used for very high and low total energy intake have previously been shown to perform equally well in identifying implausible total energy intakes compared with other more advanced methods^(32)^. Furthermore, fifty-two participants reporting > 10 energy percent (E%) from alcohol and one participant with missing data for all biomarkers of interest were excluded list-wise, leaving 1928 participants eligible for analysis. A flow chart depicting the participant flow is provided in online Supplementary Fig. 1.

This study was conducted according to the guidelines in the Declaration of Helsinki, and all procedures involving patients were approved by the Regional Committee for Medical Research Ethics (2010/267/REK West), the Norwegian Medicines Agency and the Data Inspectorate. Written informed consent was obtained from all patients.

### Dietary assessment

Dietary intake was assessed by administering a 169-item semi-quantitative FFQ developed at the Department of Nutrition, University of Oslo^(33,34)^. Participants received the FFQ at baseline visit and returned it by mail or at the one-month follow-up visit. The FFQ was designed to assess habitual food intake in the Norwegian adult population for the prior year. Frequency of consumption was collected per day, week or month depending on the food item, and portion sizes were reported as household measures. Daily intakes of food and nutrients were calculated by using a software system developed at the Department of Nutrition, University of Oslo, which is based on the Norwegian food composition table (Kostberegningssystem, version 3·2, University of Oslo, Norway). The FFQ has previously been evaluated towards weighed food records for the intake of energy, macronutrients, fatty acids and riboflavin^(33)^, which showed that the intake of energy, protein, total fat and PUFA measured by the FFQ and the weighed food records did not differ significantly. Dietary exposure variables of interest in the current study were reported intake of carbohydrate, fat and protein.

### Biochemical analyses

Blood samples (35 % fasting) were collected at baseline and stored at –80°C until analysed. Routine biochemical analyses were conducted on fresh blood samples at the laboratories in the recruiting hospitals, and study-specific analyses were performed by Bevital AS, Bergen, Norway (http://www.bevital.no) between 2000 and 2006. All metabolites were quantified using gas or liquid chromatography coupled with tandem mass spectrometry, with the exception of folate and cobalamin, which were analysed by microbiological assay^(35–39)^. Outcome variables of interest were related to the methionine-homocysteine cycle (methionine and tHcy), the transsulfuration pathway (cystathionine and cysteine), the choline oxidation pathway (choline, betaine, DMG, sarcosine, glycine and serine) and markers of related B-vitamins (riboflavin, nicotinamide (NAM), methylnicotinamide (mNAM), pyridoxal, pyridoxal 5-phosphate (PLP), pyridoxic acid, PA-ratio (PAr), folate, cobalamin and MMA). The within-day CV was 4 % for both cobalamin and folate, 3 % for PLP, 6 % for riboflavin, 1 % for tHcy and 2 % for MMA and ranged from 1 % to 2 % for cysteine, methionine, serine, glycine, cystathionine and sarcosine and 3 % to 6 % for choline, betaine and DMG. The between-day CV was 5 % for both cobalamin and folate and ranged from 6 % to 8 % for PLP and riboflavin, 2 % for tHcy, 3 % for MMA and 2 % to 4 % for cysteine, methionine, serine, glycine and cystathionine, and 3 % to 6 % for choline, betaine and DMG.

### Statistical methods

Characteristics of the study cohort are shown as geometric mean (gMean), and the 95 % prediction interval characterised by the gMean and the geometric standard deviation (gMean/gSD^1·96^, gMean × gSD^1·96^) for continuous variables and counts (%) for categorical variables. Dietary variables were energy-adjusted using the density method and expressed as E% or g/1000 kcal.

Partial Pearson correlation analyses adjusted for reported energy intake was used to assess the relationship between the dietary composition of macronutrients and the intake of different food groups, e.g. fruit and berries, grains and meat expressed as g/1000 kcal.

Associations between macronutrient intake and plasma metabolite concentrations were assessed by linear regression. Model 1 was adjusted for reported energy intake, and model 2 was further adjusted for age, sex, BMI, smoking and alcohol intake (E%). Confounding variables were identified a priori, based on current subject matter literature, using a directed acyclic graph approach. Metabolite concentrations were log-transformed before analysis, and the regression coefficients were subsequently back-transformed to provide estimates of the % change in the response variable per 1 E% increase in the exposure nutrient, accompanied by their 95 % CI. As the models are adjusted for total energy intake, an implicit concomitant isocaloric decrease of another unspecified nutrient is assumed^(40)^. The continuous associations were explored visually adjusted for Model 2 covariates, and the uncertainty was visualised by plotting hypothetical associations from bootstrapped samples (*n* 25).

Finally, we performed substitution models by modelling the specific substitutions between the macronutrients, e.g. by increasing protein intake while simultaneously reducing either carbohydrate or fat intake^(41)^. In nutritional epidemiologic research, the use of substitution models has become more prevalent^(42)^, in part because they can mimic feeding studies that modify macronutrient composition. The substitution models were adjusted for Model 2 covariates, as well as all macronutrients except the one being replaced. For example, when modelling the effect of consuming more protein at the expense of carbohydrates, protein was included in the model together with fat and total energy intake. By keeping fat and total energy intake fixed, the coefficient for protein is interpreted as the estimated effect of a 1 E% increase in protein while simultaneously reducing carbohydrate intake by 1 E%. We modelled all potential macronutrient substitutions, as well as all substitutions between SFA, MUFA and PUFA.

All statistical analyses were performed using R v3.5.1^(43)^ and the packages within the *Tidyverse* ^(44)^. The hypothetical outcome plots were generated with the *ungeviz* package^(45)^. BioRender was used to make vector graphics.

## Results

### Baseline characteristics

Baseline characteristics of the full study cohort and stratified by sex are presented in Table 1. Geometric mean (95 % prediction interval\) age was 61 (44, 85) years, BMI was 26 (20, 34) kg/m^2^ and 80 % were males.

**Table 1.** Baseline characteristics of full cohort and across sexes*

	Full cohort		Female		Male	
	*n*	%	*n*	%	*n*	%
*n*	1928		390		1538	
Male	1538	79·8 %				
Age, years						
Geometric mean	61		63·2		60·4	
95 % prediction interval	43·9, 84·8		45·2, 88·4		43·7, 83·7	
Fasting	671	34·8 %	128	32·8 %	543	35·3 %
Smoking†	559	29·0 %	109	27·9 %	450	29·3 %
Diabetes‡	592	30·7 %	117	30·0 %	475	30·9 %
Hypertension§	911	47·3 %	200	51·3 %	711	46·2 %
	Geometric mean	95 % prediction interval	Geometric mean	95 % prediction interval	Geometric mean	95 % prediction interval
Waist Circumference, cm	95·7	75·9, 120·8	88·3	66·6, 117	97·7	80·1, 119·2
BMI, kg/m	26·1	19·8, 34·4	25·8	18, 37	26·2	20·3, 33·7
CRP, mg/l	1·69	0·2, 14·43	1·82	0·21, 15·97	1·66	0·2, 14·05
eGFR, ml/min/1·73 m	88·1	59, 131·5	83·9	54, 130·4	89·2	60·6, 131·2
Methionine, µmol/l	26·5	15·6, 44·9	23·9	14·4, 39·7	27·1	16·1, 45·8
Total homocysteine, µmol/l	10·4	5·8, 18·6	9·65	5·23, 17·83	10·5	6, 18·7
Cystathionine, µmol/l	0·27	0·083, 0·86	0·24	0·076, 0·76	0·27	0·086, 0·88
Cysteine, µmol/l	286	224, 366	287	220, 375	286	224, 363
Choline, µmol/l	9·58	5·83, 15·73	9·09	5·62, 14·69	9·71	5·91, 15·94
Betaine, µmol/l	39·2	21·4, 71·6	33·2	17·2, 64·1	40·8	23·3, 71·5
DMG, µmol/l	4·05	2·15, 7·6	3·69	1·91, 7·14	4·14	2·24, 7·66
Sarcosine, µmol/l	1·51	0·76, 2·98	1·37	0·67, 2·81	1·55	0·79, 3·01
Glycine, µmol/l	203	127, 324	226	119, 428	198	133, 293
Serine, µmol/l	103	70, 150	104	68, 159	102	71, 147
Riboflavin, nmol/l	12·2	2·9, 51·6	12·8	3, 55·8	12·1	2·9, 50·6
Nicotinamide, nmol/l	363	142, 929	355	135, 936	365	144, 927
Methylnicotinamide, nmol/l	85·3	29·6, 245·6	93·5	31·5, 278·2	83·3	29·3, 236·8
Pyridoxal, nmol/l	9·88	3·59, 27·16	9·88	2·79, 34·98	9·88	3·87, 25·2
Pyridoxal 5-phosphate, nmol/l	41·6	14·4, 120·3	39·9	11·4, 139·7	42·1	15·4, 115·1
Pyridoxic acid, nmol/l	26·3	9·5, 72·5	27·4	7·8, 95·8	26	10·1, 66·9
PA-ratio	0·5	0·22, 1·1	0·54	0·25, 1·2	0·5	0·22, 1·1
Folate, nmol/l	10·6	3·6, 30·8	11·7	3·6, 37·9	10·3	3·7, 29·1
Cobalamin, pmol/l	336	144, 784	352	144, 857	332	144, 765
Methylmalonic acid, µmol/l	0·16	0·082, 0·32	0·16	0·086, 0·32	0·16	0·081, 0·32

* Continuous variables are given as geometric mean (95 % prediction interval) and categorical variables as *n* (%). CRP indicates C-reactive protein and eGFR is estimated glomerular filtration rate.

† Based on self-report and cotinine concentrations > 85 nmol/l.

‡ Diagnosed or assessed according to baseline serum glucose > 7·0 or non-fasting glucose > 11·1 mmol/l or HbA1c > 6·5.

§ Defined as preexisting diagnosis of hypertension.

**Fig. 2.**
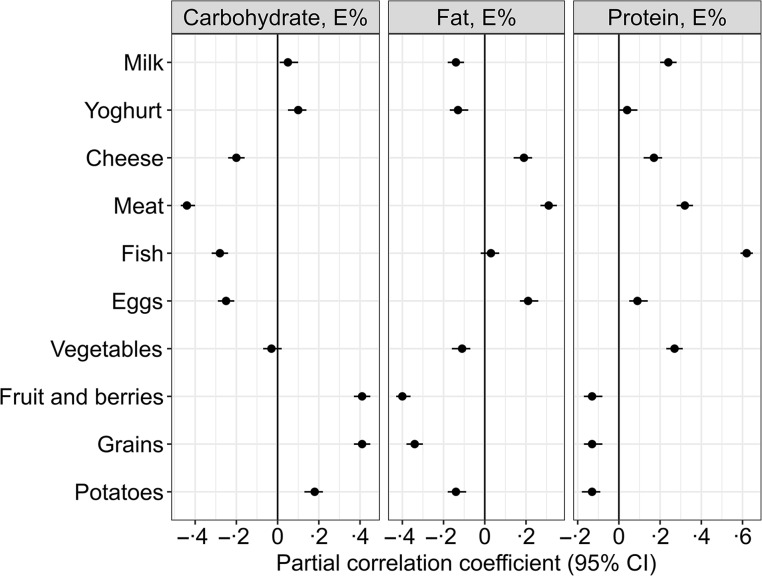
Partial Pearson correlations between the isoenergetic increases in the intake of macronutrients and the intake of different food groups (*n* 1928). Meat refers to the total intake of white and red meat, including processed meat products. The model is adjusted for reported energy intake. The intake of the different food groups, e.g. fruit and berries, grains and meat, is expressed as g/1000 kcal.

Self-reported dietary intake data are presented in Table 2. The distribution of energy intake (gMean (95 % prediction)) in the population was 49 (38, 63) E% from carbohydrate, 17 (12, 22) E% from protein and 31 (22, 45) E% from fat (of which 11 (7·3, 18) E% from SFA). Correlations between increasing proportions of total energy intake from the different macronutrients and intake of the different food groups are shown in Fig. 2.

**Table 2.** Dietary intake in full cohort and across sexes

	Full cohort		Female		Male	
	Geometric mean	95 % prediction interval	Geometric mean	95 % prediction interval	Geometric mean	95 % prediction interval
*n*	1928		390		1538	
Male						
*n*	1538					
%	79·8 %					
Energy intake, kcal	1995	1066, 3734	1548	830, 2887	2128	1217, 3721
Energy intake, kJ	8347	4460, 15 623	6477	3473, 12 079	8903	5092, 15 569
Carbohydrate, E%	49	38, 63	50	39, 64	49	37, 63
Fat, E%	31	22, 45	31	22, 44	32	22, 45
SFA, E%	11	7·3, 18	12	7·2, 18	11	7·3, 18
MUFA, E%	10	6·8, 15	9·8	6·7, 14	10	6·9, 15
PUFA, E%	7·0	4·1, 12	6·5	3·9, 11	7·1	4·2, 12
Protein, E%	17	12, 22	17	13, 23	16	12, 22
Fiber	12	7·1, 20	13	8·2, 21	12	6·9, 19
Dairy	115	17, 772	124	23, 662	113	16, 798
Meat	49	16, 152	47	19, 118	50	15, 160
Fish	45	8·7, 230	46	12, 173	44	8·1, 245
Egg	5·3	0·2, 117	5·9	0·3, 131	5·1	0·2, 113
Vegetables	84	17, 409	113	27, 469	78	16, 381
Fruit and berries	98	17, 578	122	26, 578	93	15, 566
Grains	103	52, 203	100	44, 226	104	55, 197
Potatoes	51	5·9, 446	45	3·8, 539	53	6·6, 421

E%, energy percent.

All dietary values are given as geometric mean (95 % prediction interval), and as g/1000 kcal unless otherwise noted. Dairy refers to the total intake of milk, yoghurt and cheese. Meat refers to the total intake of white and red meat, including processed meat products.

A higher carbohydrate intake was mainly associated with higher intakes of fruit and berries (*r* = 0·41), grains (*r* = 0·41) and potatoes (*r* = 0·18) and lower intakes of meat (*r* = –0·44), fish (*r* = −0·28), eggs (*r* = −0·25) and cheese (*r* = −0·2) (Fig. 2).

Higher intake of fat was associated with higher intakes of meat (*r* = 0·31), egg (*r* = 0·21) and cheese (*r* = 0·19) and lower intakes of fruit and berries (*r* = −0·4), potatoes (*r* = −0·14) and vegetables (*r* = −0·11) (Fig. 2).

Higher protein intake was associated with higher intakes of fish (*r* = 0·62), meat (*r* = 0·32), milk (*r* = 0·24), vegetables (*r* = 0·27) and cheese (*r* = 0·17) and lower intakes of fruit and berries (*r* = −0·13), grains (*r* = −0·13) and potatoes (*r* = −0·13) (Fig. 2).

### Associations between macronutrient intakes and one-carbon metabolites

Point estimates (% change (95 % CI)) for the associations between macronutrient intakes and plasma concentrations of metabolites related to one-carbon metabolism per increment of 1 E% of the exposure nutrient are shown in Table 3. Protein showed the strongest association with the outcome metabolites. Each isoenergetic increment in protein intake of 1 E% was associated with higher PLP (3·1 (2·1, 4·1)), cobalamin (2·9 (2·1, 3·7)), riboflavin (2·4 (1·1, 3·7)), PA (2·2 (1·3, 3·2)) and mNAM (2·1 (1·1, 3·1)) and lower tHcy (–1·4 (–1·9, −0·9)) and MMA (–1·4 (–2·0, −0·8)). Less strong associations were observed for other metabolites, such as sarcosine (1·0 (0·3, 1·6)), methionine (1·0 (0·5, 1·4)), glycine (–0·9 (–1·3, −0·5)), DMG (–0·7 (–1·3, −0·1)) and PAr (–0·7 (–1·4, 0·1)).

**Table 3.** Association between dietary intake and outcome metabolites*

	Carbohydrate				Fat				Protein			
	Model 1†		Model 2‡		Model 1†		Model 2‡		Model 1†		Model 2‡	
	% change	95 % confidence interval	% change	95 % confidence interval	% change	95 % confidence interval	% change	95 % confidence interval	% change	95 % confidence interval	% change	95 % confidence interval
Methionine, µmol/l	–0·1	–0·3, 0·1	–0·1	–0·3, 0·1	–0·1	–0·4, 0·1	–0·1	–0·3, 0·1	0·9	0·4, 1·4	1·0	0·5, 1·4
Homocysteine, µmol/l	0·1	–0·1, 0·3	–0·0	–0·2, 0·2	0·3	0·1, 0·5	0·3	0·1, 0·5	–1·6	–2·1, −1·1	–1·4	–1·9, −0·9
Cystathionine, µmol/l	0·4	–0·0, 0·8	0·1	–0·3, 0·6	–0·3	–0·7, 0·2	–0·2	–0·7, 0·3	0·3	–0·8, 1·3	0·3	–0·8, 1·3
Cysteine, µmol/l	0·1	–0·0, 0·1	0·0	–0·1, 0·1	–0·0	–0·1, 0·1	–0·0	–0·1, 0·1	0·0	–0·2, 0·3	0·1	–0·2, 0·3
Choline, µmol/l	0·1	–0·1, 0·3	0·0	–0·1, 0·2	–0·0	–0·2, 0·2	0·0	–0·2, 0·2	–0·5	–1·0, −0·1	–0·4	–0·8, 0·0
Betaine, µmol/l	0·4	0·2, 0·7	0·3	0·1, 0·6	–0·5	–0·7, −0·2	–0·4	–0·6, −0·1	–0·6	–1·1, −0·1	–0·1	–0·6, 0·4
DMG, µmol/l	0·1	–0·1, 0·4	0·0	–0·2, 0·3	0·2	–0·1, 0·4	0·1	–0·1, 0·4	–0·9	–1·4, −0·3	–0·7	–1·3, −0·1
Sarcosine, µmol/l	–0·1	–0·4, 0·1	–0·1	–0·4, 0·2	–0·1	–0·4, 0·1	–0·1	–0·4, 0·2	0·6	0·0, 1·3	1·0	0·3, 1·6
Glycine, µmol/l	0·4	0·2, 0·6	0·2	0·0, 0·4	–0·1	–0·3, 0·1	–0·0	–0·2, 0·1	–1·2	–1·6, −0·7	–0·9	–1·3, −0·5
Serine, µmol/l	0·0	–0·1, 0·2	–0·0	–0·2, 0·1	0·0	–0·1, 0·2	0·0	–0·1, 0·2	–0·0	–0·4, 0·3	0·1	–0·3, 0·4
Riboflavin, nmol/l	0·0	–0·5, 0·5	–0·2	–0·7, 0·4	–0·5	–1·1, 0·1	–0·3	–0·9, 0·3	2·3	1·0, 3·7	2·4	1·1, 3·7
NAM, nmol/l	–0·5	–0·9, −0·2	–0·3	–0·7, 0·1	0·3	–0·1, 0·7	0·2	–0·1, 0·6	0·9	0·0, 1·7	0·5	–0·3, 1·4
mNAM, nmol/l	–1·1	–1·5, −0·7	–0·9	–1·3, −0·5	0·6	0·2, 1·0	0·6	0·2, 1·1	2·3	1·4, 3·3	2·1	1·1, 3·1
PL, nmol/l	–0·2	–0·6, 0·2	–0·2	–0·5, 0·2	–0·5	–0·9, −0·1	–0·2	–0·6, 0·2	1·4	0·5, 2·4	1·8	0·9, 2·7
PLP, nmol/l	–0·3	–0·7, 0·0	–0·2	–0·6, 0·2	–0·8	–1·2, −0·3	–0·4	–0·9, 0·0	2·8	1·8, 3·8	3·1	2·1, 4·1
PA, nmol/l	–0·1	–0·4, 0·3	–0·2	–0·6, 0·2	–0·5	–1·0, −0·1	–0·3	–0·7, 0·1	1·9	0·9, 2·8	2·2	1·3, 3·2
PAr	0·3	–0·0, 0·6	0·0	–0·3, 0·3	0·2	–0·2, 0·5	0·1	–0·2, 0·4	–0·7	–1·4, 0·1	–0·7	–1·4, 0·1
Folate, nmol/l	–0·3	–0·7, 0·1	–0·3	–0·7, 0·2	–0·4	–0·9, 0·0	–0·2	–0·6, 0·3	1·9	1·0, 2·9	2·1	1·2, 3·1
Cobalamin, pmol/l	–0·1	–0·4, 0·2	–0·2	–0·5, 0·2	–0·4	–0·8, −0·1	–0·4	–0·8, −0·1	2·8	2·0, 3·6	2·9	2·1, 3·7
MMA, µmol/l	0·3	0·1, 0·6	0·1	–0·2, 0·3	0·2	–0·1, 0·4	0·2	–0·0, 0·5	–1·7	–2·3, −1·1	–1·4	–2·0, −0·8

DMG, dimethylglycine; MMA, methylmalonic acid; mNAM, methylnicotinamide; NAM, nicotinamide; PA, pyridoxic acid; PAr, PAr-index; PL, Pyridoxal; PLP, Pyridoxal 5’-phosphate.

* Estimates are given as % change (95 % confidence interval) in the outcome metabolite per isoenergetic increment of 1 E% in the exposure nutrient.

† Model 1 is adjusted for reported energy intake.

‡ Model 2 is adjusted for reported energy intake, alcohol intake (E%), age, sex, BMI and smoking.

**Fig. 3.**
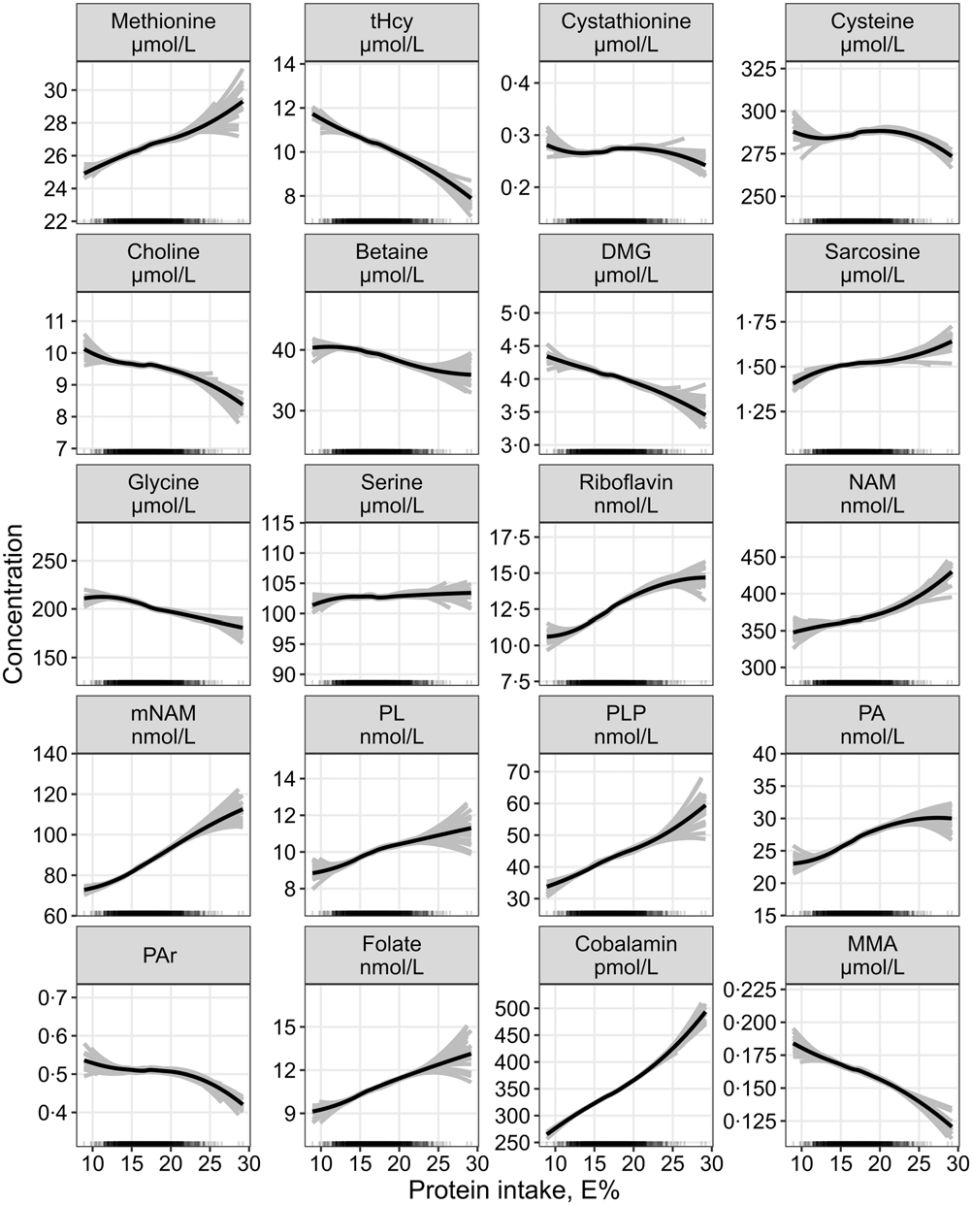
The continuous association between protein intake and plasma concentrations of one-carbon metabolites and markers of B-vitamin status assessed by linear regression, adjusted for age, sex, BMI, alcohol intake and total energy intake (*n* 1928). Metabolite concentrations were log-transformed before analysis and back-transformed to provide estimates of the % change in the response variable per 1 E% increase in the exposure nutrient. The grey lines represent hypothetical associations from twenty-five bootstrapped samples of the data, illustrating uncertainty. DMG, dimethylglycine; MMA, methylmalonic acid; mNAM, methylnicotinamide; NAM, nicotinamide; PA, pyridoxic acid; PL, pyridoxal; PLP, pyridoxal 5’-phosphate; PAr, PA-ratio; tHcy, total homocysteine.

The continuous associations between protein intake and all outcome metabolites are summarised in Fig. 3.

The strongest association observed per isoenergetic increment of carbohydrate and fat intake of 1 E % was with mNAM, which was lower with increasing carbohydrate (–0·9 (–1·3, −0·5)) and higher with increasing fat intake (0·6 (0·2, 1·1)). The continuous associations with increasing carbohydrate or fat intake are shown in online Supplementary Fig. 2 and Fig. 3, respectively.

### Substitution analyses

The substitution analyses revealed associations of similar strength regardless of whether protein replaced either carbohydrate or fat (online Supplementary Table 1), with the strongest associations observed for higher PLP (3·1 (2·2, 4·1) and 3·6 (2·5, 4·7) for carbohydrate and fat, respectively), cobalamin (2·9 (2·1, 3·7) and 3·4 (2·5, 4·3)) and riboflavin (2·4 (1·1, 3·8) and 2·7 (1·2, 4·2)). Modelling the substitution of fat with carbohydrate yielded only weak associations, with the largest effects being increased PLP (0·5 (0·0, 0·9)) and riboflavin (0·3 (–0·3, 0·9)) and decreased mNAM (–0·6 (–1·1, −0·2)).

The observed associations when substituting between different fatty acids are shown in Table 4. The strongest associations were observed when SFA replaced MUFA or PUFA, with higher riboflavin (5·0 (0·9, 9·3) and 3·3 (1·1, 5·6) for MUFA and PUFA, respectively) as well as lower PLP (–2·5 (–5·3, 0·3) and −2·7 (–4·2, −1·2)), pyridoxal (–2·5 (–5·1, 0·3) and −2·6 (–4·0, −1·1)) and folate (–2·2 (–5·0, 0·7) and −2·1 (–3·6, −0·5)). Further, replacing MUFA or PUFA with SFA was associated with higher plasma tHcy (2·3 (0·7, 3·8) and 1·3 (0·5, 2·2), respectively) and MMA (2·0 (0·2, 3·9) and 1·7 (0·7, 2·7)).

**Table 4.** Substitution analyses for the associations between different dietary fatty acid classes*

	MUFA substituting				PUFA substituting				SFA substituting			
	PUFA		SFA		MUFA		SFA		MUFA		PUFA	
	% change	95 % confidence interval	% change	95 % confidence interval	% change	95 % confidence interval	% change	95 % confidence interval	% change	95 % confidence interval	% change	95 % confidence interval
Methionine, µmol/l	–2·5	–4·0, −0·9	–2·5	–4·0, −0·9	1·9	0·4, 3·4	–0·1	–0·9, 0·8	1·7	0·3, 3·2	0·0	–0·8, 0·7
Homocysteine, µmol/l	–0·9	–2·5, 0·7	–2·2	–3·9, −0·6	1·0	–0·6, 2·6	–1·3	–2·2, −0·5	2·3	0·7, 3·8	1·3	0·5, 2·2
Cystathionine, µmol/l	–2·8	–6·1, 0·6	–2·5	–5·9, 1·1	2·2	–1·2, 5·6	0·3	–1·5, 2·2	1·6	–1·6, 4·9	–0·4	–2·1, 1·3
Cysteine, µmol/l	–1·0	–1·6, −0·3	–0·8	–1·4, −0·1	0·8	0·2, 1·5	0·2	–0·2, 0·6	0·6	–0·1, 1·2	–0·2	–0·5, 0·1
Choline, µmol/l	–0·7	–2·1, 0·7	–0·6	–2·0, 0·9	0·7	–0·6, 2·1	0·2	–0·6, 0·9	0·5	–0·8, 1·9	–0·1	–0·9, 0·6
Betaine, µmol/l	–0·6	–2·3, 1·2	0·8	–1·0, 2·6	–0·3	–1·9, 1·4	1·3	0·3, 2·2	–1·6	–3·2, −0·0	–1·4	–2·3, −0·6
DMG, µmol/l	–1·8	–3·6, 0·0	–1·2	–3·0, 0·7	1·1	–0·6, 2·9	0·6	–0·4, 1·6	0·4	–1·3, 2·1	–0·7	–1·6, 0·2
Sarcosine, µmol/l	–1·6	–3·6, 0·4	–0·1	–2·1, 2·0	0·6	–1·3, 2·6	1·4	0·4, 2·5	–0·9	–2·8, 0·9	–1·6	–2·6, −0·6
Glycine, µmol/l	–0·0	–1·3, 1·3	0·5	–0·8, 1·9	–0·2	–1·4, 1·0	0·5	–0·2, 1·2	–0·7	–1·9, 0·5	–0·6	–1·2, 0·1
Serine, µmol/l	0·1	–1·0, 1·2	0·2	–1·0, 1·4	–0·4	–1·5, 0·7	0·0	–0·6, 0·7	–0·5	–1·6, 0·5	–0·1	–0·7, 0·4
Riboflavin, nmol/l	–0·2	–4·4, 4·1	–3·2	–7·5, 1·2	2·0	–2·1, 6·3	–2·8	–5·0, −0·5	5·0	0·9, 9·3	3·3	1·1, 5·6
NAM, nmol/l	0·2	–2·6, 3·0	0·1	–2·8, 3·0	0·3	–2·4, 3·0	–0·1	–1·6, 1·5	0·4	–2·2, 3·1	0·2	–1·2, 1·6
mNAM, nmol/l	–0·2	–3·3, 2·9	1·8	–1·5, 5·2	1·0	–2·0, 4·1	2·2	0·4, 3·9	–1·0	–3·8, 2·0	–1·8	–3·3, −0·2
PL, nmol/l	–0·6	–3·5, 2·4	2·1	–1·0, 5·3	0·1	–2·7, 3·0	2·6	1·0, 4·3	–2·5	–5·1, 0·3	–2·6	–4·0, −1·1
PLP, nmol/l	–0·9	–3·9, 2·2	1·9	–1·3, 5·2	0·1	–2·8, 3·1	2·7	1·0, 4·4	–2·5	–5·3, 0·3	–2·7	–4·2, −1·2
PA, nmol/l	–0·7	–3·6, 2·2	1·9	–1·1, 5·1	0·8	–2·1, 3·7	2·7	1·1, 4·4	–1·8	–4·5, 0·9	–2·5	–3·9, −1·0
PAr	0·1	–2·2, 2·5	0·0	–2·4, 2·5	0·6	–1·6, 2·9	0·0	–1·3, 1·3	0·7	–1·5, 3·0	0·2	–1·0, 1·4
Folate, nmol/l	–0·1	–3·2, 3·0	2·0	–1·2, 5·4	–0·2	–3·1, 2·9	2·1	0·4, 3·9	–2·2	–5·0, 0·7	–2·1	–3·6, −0·5
Cobalamin, pmol/l	–2·0	–4·4, 0·5	–0·6	–3·1, 2·0	1·3	–1·1, 3·7	1·3	–0·0, 2·7	–0·2	–2·5, 2·2	–1·4	–2·6, −0·1
MMA, µmol/l	–0·3	–2·2, 1·7	–2·0	–3·9, −0·0	0·4	–1·5, 2·2	–1·7	–2·7, −0·7	2·0	0·2, 3·9	1·7	0·7, 2·7

DMG, dimethylglycine; MMA, methylmalonic acid; mNAM, methylnicotinamide; NAM, nicotinamide; PA, pyridoxic acid; PAr, PAr-index; PL, pyridoxal; PLP, pyridoxal 5’-phosphate.

* Estimates are given as % change (95 % CI) in the outcome metabolite per isoenergetic substitution of 1 E% in the exposure nutrient for the replacement nutrient. The model is adjusted for reported energy intake, age, sex, BMI, smoking and the non-substituted nutrients.

## Discussion

In patients with stable angina pectoris, we observed that self-reported intakes of protein, carbohydrate and fat were associated with circulating concentrations of metabolites related to one-carbon metabolism and related B-vitamin cofactors. The strongest associations were observed between increasing protein intake and higher plasma concentrations of PLP, cobalamin, riboflavin and mNAM and lower tHcy and MMA. Our observations did not appear to be affected by whether protein replaced carbohydrate or fat. Moreover, dietary fatty acid composition was associated with plasma concentrations of several biomarkers, most notably higher plasma concentrations of riboflavin, tHcy and MMA, as well as lower pyridoxal, PLP and folate when SFA replaced MUFA or PUFA.

### Possible mechanisms

The mechanisms underlying our observations may be directly related to dietary composition and differences in intakes of one-carbon metabolites and B-vitamins or indirectly due to potential metabolic alterations in response to dietary intake.

Higher protein intake was positively associated with the intake of fish and meat, which are major sources of niacin, vitamin B_6_, folate and cobalamin, as well as with dairy products, which are major dietary sources of riboflavin. These food items may have directly contributed to the higher plasma concentrations of these B-vitamins. Higher concentrations of vitamin B_6_ markers following increased protein intake are consistent with the literature^(21)^. This is also reflected by a slight yet linear decrease in PAr in our study, which could indicate lower cellular inflammation, as a high PAr has been associated with increased systemic inflammation and vitamin B_6_ catabolism^(46)^. Several studies have also shown that increased intakes of folate, vitamin B_6_ and cobalamin lower plasma tHcy^(47–49)^, which was also seen with increasing protein intake in the present study. Further, we observed higher plasma concentrations of cobalamin and lower plasma concentrations of MMA with increasing protein intake. During a cobalamin-deficient state, the cobalamin-dependent enzyme methylmalonyl-CoA mutase, which catalyses the formation of succinyl-CoA from methylmalonyl-CoA is inhibited, leading to an accumulation of MMA as an alternate mechanism. MMA is thus regarded as a functional marker of cobalamin status. The observation that increased plasma concentration of cobalamin is simultaneously observed with lower plasma concentration of MMA is therefore expected^(50)^.

Higher protein intake in our study was also associated with higher methionine and lower plasma tHcy concentrations, the latter being in line with a prior report investigating dietary factors associated with tHcy in an elderly population^(51)^. Previously, protein restriction has been reported to increase the partitioning of homocysteine towards remethylation as a means of conserving methionine^(15,16,52)^. Excess methionine intake, however, was shown to reduce remethylation and increase homocysteine catabolism through the transsulfuration pathway^(53)^. This may be related to methionine being a precursor for the universal methyl donor, S-adenosylmethionine, which inhibits remethylation and stimulates transsulfuration^(54)^. Further, plasma tHcy concentrations are believed to be highly dependent on the rate of synthesis during transmethylation reactions, and endogenous production of creatine and phosphatidylcholine are generally thought to be the main metabolic sources of plasma tHcy^(55)^. Higher dietary intakes of preformed creatine and choline, of which the main dietary sources are animal foods, such as meat, fish and eggs, could consequently reduce the requirement for their endogenous synthesis, limiting homocysteine production. However, increased cellular S-adenosylmethionine concentrations stimulate glycine-N-methyltransferase (GNMT) in the liver, which catalyses an S-adenosylmethionine-dependent methylation of glycine forming sarcosine and S-adenosylhomocysteine, the precursor of homocysteine (Fig. 1). The GNMT reaction has been suggested to be a key regulator of cellular methylation status^(56)^. Scavenging of excess methyl groups through GNMT is consistent with the observed inverse association between protein intake and glycine concentrations, as well as the positive association with sarcosine. Together, a reduced demand for choline and creatine synthesis could possibly counteract the tHcy elevating effect of increased GNMT flux.

A higher protein intake was also associated with higher concentrations of plasma folate, while for a higher intake of carbohydrate (online Supplementary Fig. 2), fat (online Supplementary Fig. 3) and SFA, the opposite was observed. Interestingly, as protein-rich foods in general are not the main dietary sources of folate, the increase in plasma folate may be indirect and result from a metabolic response to increased protein intake. For instance, it could be related to other factors such as increased intake and/or availability of the cofactors riboflavin, NADH and NADPH, necessary for the conversion of 5-methylenetetrahydrofolate to mTHF by the MTHFR enzyme, as a higher protein intake also demonstrated higher plasma concentrations of these metabolites.

Although we did not measure peroxisome proliferator-activated receptor (PPAR) *α* activity in the current study, it could be speculated that some of the observed associations, in particular those estimated with changing fat composition, may be partly mediated through altered PPAR*α* activity. The nuclear receptor PPAR*α* is a central nutritional sensor and regulator of energy metabolism^(57)^. Evidence from both rodent and human studies has linked PPAR*α* to transcriptional regulation of several key enzymes in the one-carbon metabolism pathways, including downregulation of GNMT and both enzymes in the transsulfuration pathway, leading to altered metabolite concentrations in plasma^(58–66)^. PPAR*α* activation has also been linked to circulating markers of B-vitamin status, such as higher concentrations of niacin^(58,63,66)^, vitamin B_6_^(58,66,67)^ and MMA^(58,66)^. Among other mechanisms, PPAR*α* is activated by dietary fatty acids, particularly PUFA^(68,69)^. Others have noted that the amount and composition of dietary fatty acids may influence PPAR*α* activity^(70)^.

### Clinical implications

Diet is an important modifiable lifestyle factor, and a role in the regulation of one-carbon metabolism could potentially mediate a link between diet and CVD risk. The observations reported in the current study suggest that protein intake could have a prominent role in the regulation of one-carbon metabolism. Moreover, the metabolic phenotype observed with increasing protein intake (lower tHcy, DMG and MMA and higher concentration of B-vitamin status markers) could be considered beneficial regarding CVD risk. As noted, some studies suggest that high protein animal diets and high protein plant diets have opposite effects, where the first is positively associated with tHcy and the latter inversely associated with tHcy^(19,20)^. Suggested explanations for this include that high-protein plant diets contain more folate compared with high protein animal diets, which serves as a cofactor in the remethylation pathway of tHcy to methionine, thus reducing tHcy concentrations. While our study did not distinguish between protein of different animal and plant origin, future studies should incorporate this differentiation. Additionally, the observational nature of our study necessitates caution in drawing definitive conclusions, and clinical studies investigating the direct effects of increasing protein intake on concentrations of CVD risk-associated metabolites are needed before recommending an increased protein consumption. Furthermore, our observations revealed lower plasma concentrations of glycine with increasing protein intake, an amino acid previously associated with increased risk of acute myocardial infarction and type 2 diabetes^(71,72)^. This underscores the need for caution and prompts further research to thoroughly explore the nuanced effects of increased protein intake on CVD risk.

In healthy subjects with moderate hypercholesterolaemia, we previously reported that changing dietary fat composition by replacing SFA with PUFA influenced circulating concentrations of one-carbon metabolites and B-vitamins^(23)^. The observations for fat types in the current study, when modelling the same substitution, were largely consistent with what we previously reported. Given the central role of PPAR*α* in the regulation of energy and lipid metabolism^(57,73)^, biomarkers reflecting endogenous PPAR*α*-activity may be of interest when considering CVD risk, as well as individually tailored dietary advice. We and others have proposed pathway-linked metabolites as potential biomarkers of PPAR*α*-activity, including NAM, mNAM, pyridoxal, DMG and MMA^(58,74–76)^. Taken together with our previous findings, further studies are needed to clarify to what extent circulating concentrations of one-carbon metabolites are modulated through dietary influences on PPAR*α* activation.

### Strengths and limitations

The main strength of these analyses is the use of a large and well-characterised study population, with comprehensive information on baseline characteristics allowing us to control for a wide variety of potential confounding factors.

Several limitations also merit attention. First, the cross-sectional design does not allow causal inference regarding the temporal effects of dietary composition on plasma concentrations of one-carbon metabolites and markers of B-vitamin status. Second, the metabolites discussed in this paper are partly influenced by factors other than diet. Although we controlled for the most important factors, such as age, sex, BMI, smoking and alcohol intake, we cannot exclude the potential for residual confounding. Third, the prandial state at the time of blood sampling varied among participants, with 34·8 % considered fasting. It is widely acknowledged that prandial status can influence the circulating concentrations of metabolites examined in this study^(77,78)^. However, prandial status at baseline is unrelated to the exposure, namely the dietary composition of the individuals, and therefore we do not consider fasting status a confounder for the associations explored between dietary composition and metabolite concentrations. Consequently, it was not included in the statistical models. Fourth, self-reported dietary data come with inherent measurement error. It is known that FFQ-derived data are affected by systematic errors^(79)^, meaning the reported intakes must be interpreted with caution and cannot be taken at face value. However, FFQ data are suited to rank individuals according to their estimated average dietary intakes, allowing for estimating associations between habitual diet and outcome. As measurement errors for the individual nutrients are highly correlated with the measurement error in reported total energy intake, energy-adjusted estimates, such as nutrient densities (e.g. E% or g/1000 kcal), correspond better with true intakes^(79)^. Furthermore, adjusting the regression models for self-reported energy intakes increases the precision of the estimates^(80)^. It can be assumed that the measurement error in dietary intake data is non-differential, meaning that the overall effect is on average expected to attenuate the ‘true’ associations due to regression dilution bias^(81)^. The FFQ used in this study was designed to capture the habitual diet during the past year, and consequently, temporal changes of shorter duration may be missed. Thus, we cannot comment on short-term effects of diet. Fifth, we did not differentiate between different protein sources, including subtypes of animal and plant sources, which could be of importance. Finally, the population consisted of mostly male patients above 60 years of age with established CVD, limiting the generalisability of our observations. Nonetheless, our metabolite analyses of a relatively large sample add to our knowledge of how macronutrient intake interacts with plasma metabolite markers, which may guide future studies of how altered macronutrient composition influences CVD risk.

### Conclusion

Our observations in this population of patients with stable angina pectoris suggest that dietary macronutrient composition influence plasma concentration of one-carbon metabolites and markers of B-vitamin status. A higher protein intake, as well as replacing SFA with MUFA and PUFA, was associated with a more favourable metabolic phenotype regarding metabolites associated with CVD risk. Future studies should assess whether the observed associations mirror an effect of macronutrients and whether source of protein is of importance.

## Supporting information

Bråtveit et al. supplementary material 1 Bråtveit et al. supplementary material

Bråtveit et al. supplementary material 2 Bråtveit et al. supplementary material

Bråtveit et al. supplementary material 3 Bråtveit et al. supplementary material

Bråtveit et al. supplementary material 4 Bråtveit et al. supplementary material

## Supplementary material

For supplementary material/s referred to in this article, please visit https://doi.org/10.1017/S0007114524000473
