# Supplementary material for: Association between dietary macronutrient composition and plasma one-carbon metabolites and B-vitamin cofactors in patients with stable angina pectoris
Source: Br J Nutr. 2024 Feb 16;131(10):1678–90. doi: 10.1017/S0007114524000473 (PMC11063666; doi:10.1017/S0007114524000473)
Supplement: Bråtveit et al. supplementary material 1 — Bråtveit et al. supplementary material [file S0007114524000473sup001.pdf]

Supplementary data

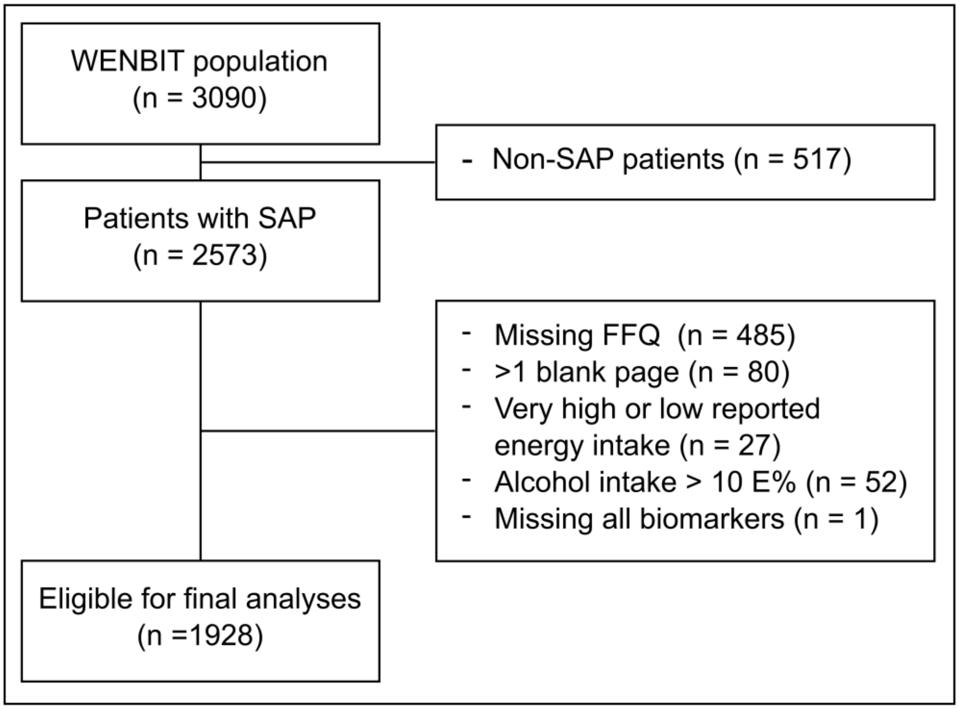


**Supplementary Figure 1**. Flow chart showing the selection of participants eligible for the study.
