# Supplementary material for: Association between dietary macronutrient composition and plasma one-carbon metabolites and B-vitamin cofactors in patients with stable angina pectoris
Source: Br J Nutr. 2024 Feb 16;131(10):1678–90. doi: 10.1017/S0007114524000473 (PMC11063666; doi:10.1017/S0007114524000473)
Supplement: Bråtveit et al. supplementary material 2 — Bråtveit et al. supplementary material [file S0007114524000473sup002.pdf]

Supplementary Table 1: Substitution analyses for the associations between dietary intake and outcome metabolites[1]

	Carbohydrate substituting		Fat substituting		Protein substituting	
	Fat	Protein	Carbohydrate	Protein	Carbohydrate	Fat
Methionine	0.1 (-0.1, 0.4)	-1.0 (-1.4, -0.5)	-0.1 (-0.3, 0.1)	-1.0 (-1.6, -0.5)	1.0 (0.5, 1.5)	1.1 (0.6, 1.6)
Homocysteine	-0.3 (-0.6, -0.1)	1.4 (0.9, 1.9)	0.3 (0.1, 0.5)	1.7 (1.1, 2.2)	-1.4 (-1.9, -0.9)	-1.7 (-2.3, -1.2)
Cystathionine	0.2 (-0.3, 0.7)	-0.3 (-1.3, 0.8)	-0.2 (-0.7, 0.3)	-0.5 (-1.6, 0.7)	0.3 (-0.8, 1.3)	0.5 (-0.7, 1.7)
Cysteine	0.0 (-0.1, 0.1)	-0.1 (-0.3, 0.2)	0.0 (-0.1, 0.1)	-0.1 (-0.3, 0.2)	0.1 (-0.2, 0.3)	0.1 (-0.2, 0.3)
Choline	-0.0 (-0.2, 0.2)	0.4 (-0.0, 0.9)	0.0 (-0.2, 0.2)	0.5 (-0.0, 0.9)	-0.4 (-0.9, 0.0)	-0.5 (-0.9, 0.0)
Betaine	0.4 (0.1, 0.6)	0.1 (-0.4, 0.6)	-0.4 (-0.6, -0.1)	-0.3 (-0.9, 0.3)	-0.1 (-0.6, 0.4)	0.3 (-0.3, 0.9)
DMG	-0.1 (-0.4, 0.1)	0.7 (0.1, 1.3)	0.1 (-0.1, 0.4)	0.8 (0.2, 1.4)	-0.7 (-1.3, -0.1)	-0.8 (-1.4, -0.2)
Sarcosine	0.1 (-0.2, 0.4)	-1.0 (-1.6, -0.4)	-0.1 (-0.4, 0.2)	-1.0 (-1.7, -0.4)	1.0 (0.4, 1.6)	1.1 (0.4, 1.8)
Glycine	0.0 (-0.1, 0.2)	0.9 (0.5, 1.3)	0.0 (-0.2, 0.1)	0.8 (0.4, 1.2)	-0.9 (-1.3, -0.5)	-0.8 (-1.3, -0.4)
Serine	-0.0 (-0.2, 0.1)	-0.1 (-0.4, 0.3)	0.0 (-0.1, 0.2)	-0.0 (-0.4, 0.3)	0.1 (-0.3, 0.4)	0.0 (-0.3, 0.4)
Riboflavin	0.3 (-0.3, 0.9)	-2.3 (-3.6, -1.1)	-0.3 (-0.9, 0.3)	-2.6 (-3.9, -1.2)	2.4 (1.1, 3.8)	2.7 (1.2, 4.2)
NAM	-0.3 (-0.7, 0.2)	-0.5 (-1.4, 0.3)	0.2 (-0.2, 0.6)	-0.3 (-1.2, 0.7)	0.5 (-0.3, 1.4)	0.3 (-0.7, 1.3)
mNAM	-0.6 (-1.1, -0.2)	-2.0 (-3.0, -1.1)	0.6 (0.2, 1.0)	-1.4 (-2.4, -0.4)	2.1 (1.1, 3.1)	1.5 (0.4, 2.6)
PL	0.2 (-0.2, 0.7)	-1.8 (-2.7, -0.9)	-0.2 (-0.6, 0.2)	-1.9 (-2.9, -1.0)	1.8 (0.9, 2.7)	2.0 (1.0, 3.1)
PLP	0.5 (0.0, 0.9)	-3.0 (-3.9, -2.1)	-0.4 (-0.9, 0.0)	-3.4 (-4.3, -2.4)	3.1 (2.2, 4.1)	3.6 (2.5, 4.7)
PA	0.3 (-0.1, 0.7)	-2.2 (-3.1, -1.3)	-0.3 (-0.7, 0.1)	-2.4 (-3.3, -1.4)	2.2 (1.3, 3.2)	2.5 (1.5, 3.6)
PAr	-0.1 (-0.5, 0.2)	0.7 (-0.1, 1.4)	0.1 (-0.2, 0.4)	0.7 (-0.0, 1.5)	-0.7 (-1.4, 0.1)	-0.8 (-1.6, 0.0)
Folate	0.2 (-0.3, 0.6)	-2.1 (-3.0, -1.2)	-0.2 (-0.6, 0.3)	-2.2 (-3.2, -1.2)	2.1 (1.2, 3.1)	2.3 (1.2, 3.4)
Cobalamin	0.4 (0.1, 0.8)	-2.9 (-3.6, -2.1)	-0.4 (-0.8, -0.1)	-3.2 (-4.0, -2.4)	2.9 (2.1, 3.7)	3.4 (2.5, 4.3)
MMA	-0.3 (-0.5, 0.0)	1.4 (0.8, 2.0)	0.3 (-0.0, 0.5)	1.6 (1.0, 2.3)	-1.4 (-2.0, -0.8)	-1.7 (-2.3, -1.0)

[1]Estimates are given as % change (95% confidence interval) in the outcome metabolite per isoenergetic substitution of 1 E% in the exposure nutrient for the replacement nutrient. The Model is adjusted for reported energy intake, age, sex, BMI, smoking and the non-substituted nutrient. DMG, dimethylglycine; MMA, methylmalonic acid; mNAM, methylnicotinamide; NAM, nicotinamide; PA, pyridoxic acid; PAr, PAr-index; PL, Pyridoxal; PLP, Pyridoxal-5'-phosphate
