# Supplementary material for: Association between dietary macronutrient composition and plasma one-carbon metabolites and B-vitamin cofactors in patients with stable angina pectoris
Source: Br J Nutr. 2024 Feb 16;131(10):1678–90. doi: 10.1017/S0007114524000473 (PMC11063666; doi:10.1017/S0007114524000473)
Supplement: Bråtveit et al. supplementary material 3 — Bråtveit et al. supplementary material [file S0007114524000473sup003.pdf]

Supplementary data

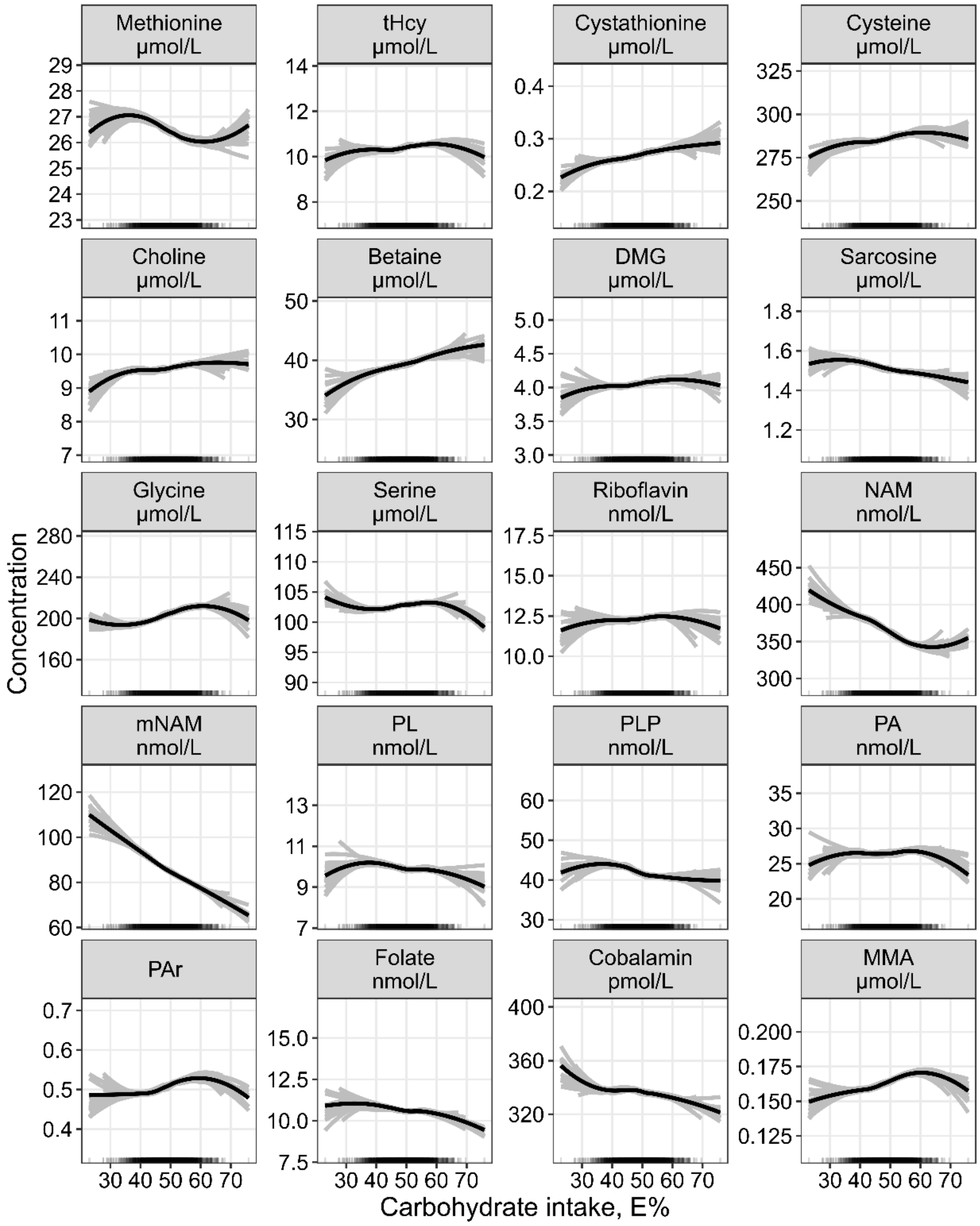

Supplementary figure 2: The continuous association between carbohydrate intake and plasma concentrations of one-carbon metabolites and markers of B-vitamin status, assessed by linear regression, adjusted for age, sex, BMI, alcohol intake, and total energy intake (n = 1928). Metabolite concentrations were log-transformed before analysis and back-transformed to provide estimates of the % change in the response variable per 1 E% increase in the exposure nutrient. The grey lines represent hypothetical associations from 25 bootstrapped samples of the data, illustrating uncertainty. DMG, dimethylglycine; MMA, methylmalonic acid; mNAM, methylnicotinamide; NAM, nicotinamide; PA, pyridoxic acid; PL, pyridoxal; PLP, pyridoxal 5'-phosphate; PAr, PA-ratio; tHcy, total homocysteine.
